# Organ‐Specific Volatile Profiling of *Lactuca tatarica* (L.) C.A.Mey. (Asteraceae) Reveals Metabolic Differentiation by MSD–SPME–GC–MS/FID

**DOI:** 10.1002/cbdv.71736

**Published:** 2026-09-20

**Authors:** Emil N. Shukurlu, Gulmira Özek, Temel Özek, Marcello Iriti, Sara Vitalini

**Affiliations:** ^1^ Institute of Botany Public Legal Entity Ministry of Science and Education of the Republic of Azerbaijan Baku Azerbaijan; ^2^ Department of Pharmacognosy Faculty of Pharmacy Anadolu University, Yunus Emre Kampüsü Tepebasi/Eskisehir Türkiye; ^3^ Medicinal Plant, Drug and Scientific Research and Application Center (AUBIBAM) Anadolu University, Yunus Emre Kampüsü Tepebasi/Eskisehir Türkiye; ^4^ Department of Biomedical, Surgical and Dental Sciences Università degli Studi di Milano Milan Italy

**Keywords:** aldehydes, Asteraceae, headspace analysis, terpenoids, VOCs

## Abstract

The organ‐specific volatile profile of *Lactuca tatarica* (L.) C.A.Mey. (roots, leaves, and seeds), collected in Azerbaijan, was investigated using microsteam distillation–solid phase microextraction (MSD–SPME) coupled with gas chromatography–mass spectrometry/flame ionization detection (GC–MS/FID). A total of 46 compounds belonging to eight major chemical classes were identified. Clear organ‐dependent differences emerged: leaves exhibited the highest chemical diversity, with 34 constituents, and were dominated by lipid‐derived aldehydes; roots (21) showed a relative enrichment in terpenoids, while seeds displayed a simpler composition (16), mainly consisting of aldehydes and aromatic ketones. The predominant ones were phenylacetaldehyde in leaves (13.3%), hexanal in roots (18.0%), and nonanal in seeds (16.3%). These results highlight distinct metabolic differentiation reflected in the volatile profiles of the three organs and contribute to the phytochemical characterization of the genus *Lactuca*.

## Graphical abstract

MSD–SPME–GC–MS/FID reveals distinct organ‐specific volatile signatures in *Lactuca tatarica*. Leaves are dominated by lipid‐derived aldehydes, roots exhibit a mixed profile of aldehydes and terpenoids, and seeds are characterized by aldehydes and aromatic ketones. These findings expand the phytochemical knowledge of this species and highlight organ‐specific metabolic differentiation.

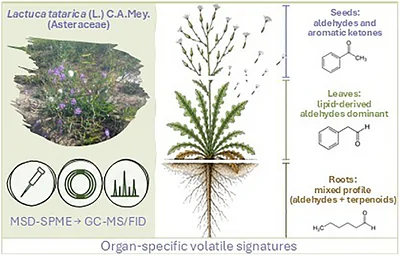

## 1 Introduction

The family Asteraceae represents one of the largest and most diverse groups of angiosperms, comprising approximately 1600–1700 genera and up to 30 000 species distributed worldwide [1]. The genus *Lactuca* L. includes about 150 species, many of which are adapted to xerophytic and semiarid environments [2, 3].

*Lactuca tatarica* (L.) C.A.Mey. is a perennial plant widely distributed in Eurasian steppe and coastal areas, particularly around the Caspian Sea, where it grows on sandy and saline soils [3, 4, 5]. It is also reported as a common ruderal species in disturbed habitats across the Caucasus region [6], indicating notable ecological adaptability. *L. tatarica* has been traditionally used in folk medicine in several Asian countries for the management of inflammatory, gastrointestinal, febrile, and musculoskeletal disorders [7, 8, 9, 10, 11, 12], and, like other members of the genus, produces a latex (lactucarium) rich in bioactive constituents associated with therapeutic properties [13].

More generally, *Lactuca* taxa are known to synthesize a wide range of secondary metabolites, reflecting their considerable chemical diversity [14]. Previous studies have mainly addressed essential oil composition and, in some cases, fatty acid profiles, thus providing only partial insight into the overall volatile fraction [15, 16, 17].

In *L. tatarica*, the available phytochemical literature has focused on non‐volatile constituents, such as sesquiterpene lactones and flavonoids [18, 19, 20, 21]. Although volatile organic compounds (VOCs) play key roles in plant physiology and ecological interactions, including processes related to environmental adaptation [22, 23], and often reflect tissue‐specific metabolic specialization [24, 25], the VOC profile of this species remains poorly characterized, particularly with regard to its distribution across different plant organs.

In addition, conventional extraction techniques, such as hydrodistillation and solvent extraction, are generally time‐consuming, often require relatively large amounts of plant material, and may also involve the use of organic solvents [26]. In this context, the combination of microsteam distillation and solid‐phase microextraction (MSD–SPME) represents an effective approach for the analysis of VOCs, enabling rapid and solvent‐free sampling at microscale levels [27, 28, 29].

Accordingly, examining VOC variation among plant organs provides valuable information on metabolic specialization and functional differentiation within plant systems. However, such investigations remain limited for many species, including *L. tatarica*, for which a comprehensive characterization is still lacking. Therefore, the present work aimed to fill this gap by determining its root‐, leaf‐, and seed‐specific VOC profile through MSD–SPME coupled with GC–MS/FID and elucidating patterns of metabolic variation among these organs.

## 2 Results and Discussion

**FIGURE 1:**
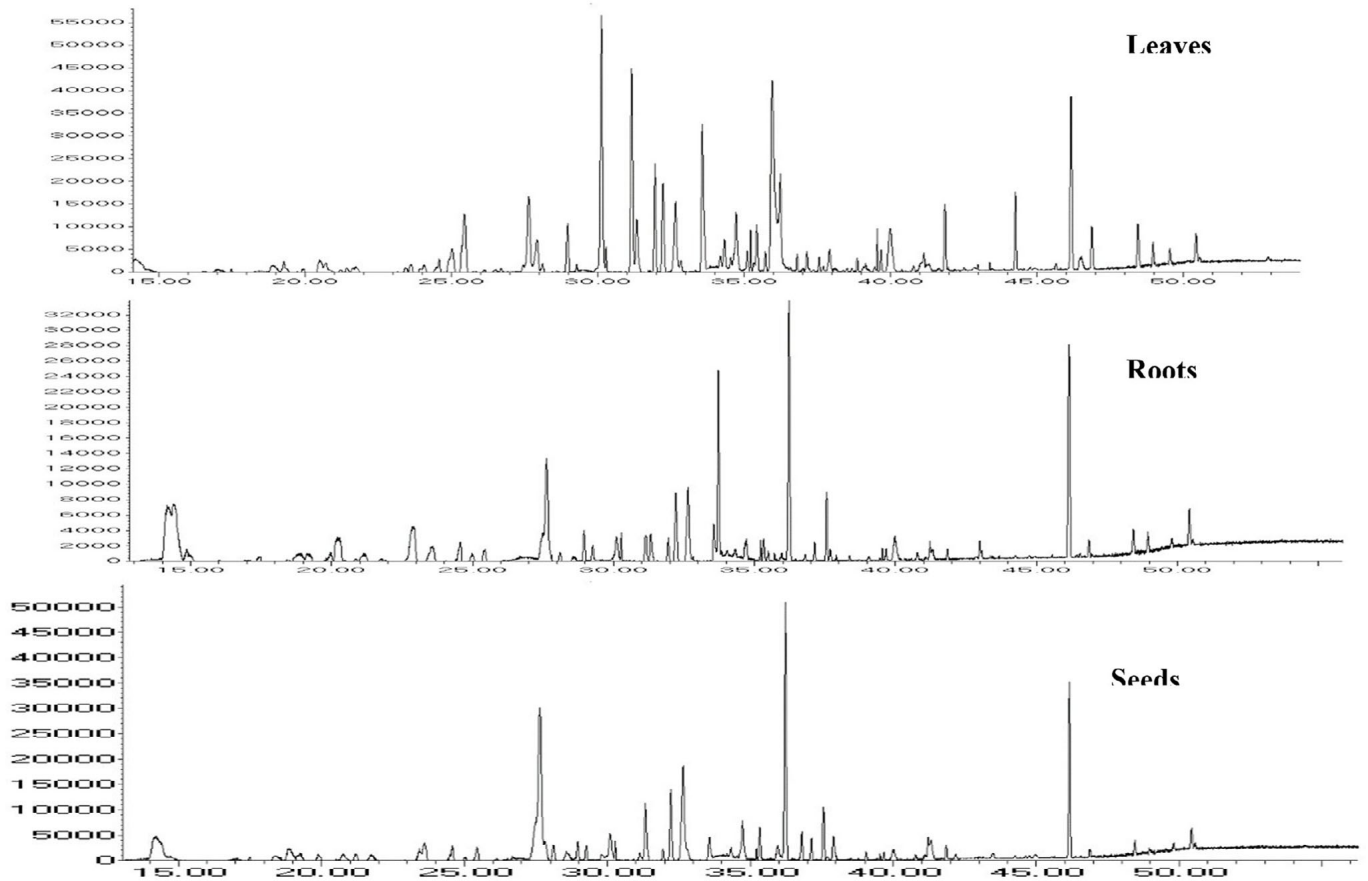
Chromatograms of the volatile profiles of *L. tatarica* leaves, roots, and seeds obtained by MSD–SPME–GC–MS/FID.

**FIGURE 2:**
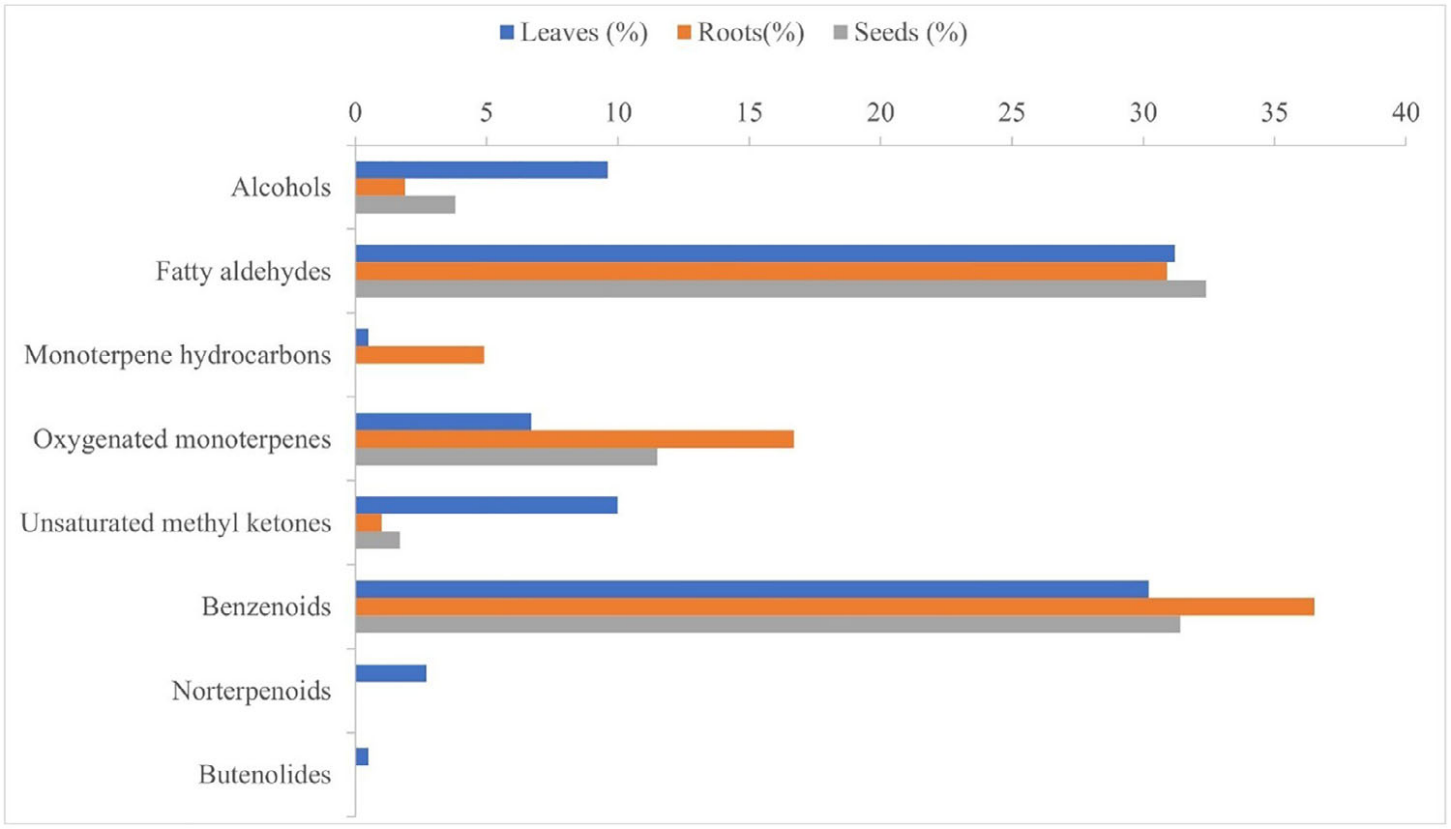
Relative distribution (%) of the major chemical classes identified in leaves, roots, and seeds of *L. tatarica* by MSD–SPME–GC–MS/FID.

A total of 46 volatile compounds were identified in *L. tatarica* leaves, roots, and seeds (Table 1 and Figure 1). Their relative distribution by major chemical classes is presented in Figure 2. Overall, the composition varied markedly across the three investigated organs. Leaves exhibited the highest number of constituents (34), followed by roots (21) and seeds (16), demonstrating clear organ‐dependent metabolic differentiation. Comparable trends have also been reported in other plant species, including members of the Asteraceae, where tissue specialization strongly influences VOC composition and distribution [15, 24, 25].

**TABLE 1:** Volatile compounds identified in *L. tatarica* leaves, roots, and seeds by GC–MS/FID.

No.	RRI^a^	RRI^b^	Compound	Leaves^c^ (%) ± SD	Roots^c^ (%) ± SD	Seeds^c^ (%) ± SD
1	1093	1056–1106 [30]	Hexanal	1.80 ± 0.08	**18.00 ± 1.2**	**6.30 ± 0.40**
**2**	1194	1163–1208 [30]	Heptanal	0.70 ± 0.04	—	—
**3**	1203	1178–1219 [30]	Limonene	0.50 ± 0.01	—	—
**4**	1213	1186–1231 [30]	1,8‐Cineole	—	3.50 ± 0.20	—
**5**	1280	1246–1291 [30]	*p*‐Cymene	—	4.90 ± 0.30	—
**6**	1296	1267–1312 [30]	Octanal	—	—	1.60 ± 0.10
**7**	1341	1289–1339 [30]	(Z)‐2‐Heptenal	1.60 ± 0.09	—	—
**8**	1348	1289–1339 [30]	6‐Methyl‐5‐hepten‐2‐one	3.60 ± 0.20	—	—
**9**	1360	1316–1377 [30]	Hexanol	—	1.90 ± 0.10	3.80 ± 0.20
**10**	1400	1370–1414 [30]	Nonanal	4.10 ± 0.45	**8.50 ± 0.50**	**16.30 ± 1.00**
**11**	1416	1415 [31]	3‐Octen‐2‐one	—	—	1.10 ± 0.00
**12**	1431	1415 [32]	4,8‐Dimethyl‐1,3,7‐nonatriene	1.80 ± 0.09	—	—
**13**	1441	1407–1463 [30]	(E)‐2‐Octenal	1.80 ± 0.21	1.70 ± 0.10	1.20 ± 0.05
**14**	1451	1409–1446 [30]	o‐Methyl anisole (=1‐methoxy‐2‐methylbenzene)	—	0.70 ± 0.03	t
**15**	1474	1474 [33]	6,10‐Dihydromyrcenol	—	—	2.60 ± 0.18
**16**	1479	1480 [34]	(E,Z)‐2,4‐Heptadienal	**10.60 ± 1.0**	—	—
**17**	1506	1471–1516 [30]	Decanal	2.20 ± 0.08	1.40 ± 0.03	4.00 ± 0.11
**18**	1507	1455–1514 [30]	(E,E)‐2,4‐Heptadienal	**7.60 ± 0.60**	1.30 ± 0.05	—
**19**	1523	1535 [35]	3,5‐Octadien‐2‐one	—	1.00 ± 0.08	0.60 ± 0.00
**20**	1535	1513 [36]	(E,Z)‐3,5‐Octadien‐2‐one	3.50 ± 0.25	—	—
**21**	1538	1534 [37]	*trans*‐Chrysanthenyl acetate	—	**8.10 ± 0.70**	—
**22**	1541	1481–1555 [30]	Benzaldehyde	3.30 ± 0.10	4.10 ± 0.30	4.70 ± 0.12
**23**	1553	1507–1564 [30]	Linalool	3.40 ± 0.23	**5.10 ± 0.22**	**8.90 ± 0.80**
**24**	1572	—	2‐*tert*‐Butylcyclohexanol	4.00 ± 0.30	—	—
**25**	1573	1576 [38]	(*E,E*)‐3,5‐Octadien‐2‐one	1.90 ± 0.08	—	—
**26**	1608	1604 [39]	6‐Methyl‐3,5‐heptadien‐2‐one	1.00 ± 0.09	—	—
**27**	1611	1582–1630 [30]	Undecanal	—	—	3.00 ± 0.10
**28**	1621	—	2‐*tert*‐Butylcyclohexanol isomer^d^	2.60 ± 0.12	—	—
**29**	1638	1548–1638 [30]	*β*‐Cyclocitral	1.50 ± 0.10	—	—
**30**	1641	1583–1656 [30]	Methyl benzoate	—	1.00 ± 0.08	—
**31**	1663	1605–1680 [30]	Phenylacetaldehyde	**13.30** ± **1.10**	—	—
**32**	1671	1607–1699 [30]	Acetophenone	4.30 ± 0.30	**13.40 ± 1.0**	**15.10 ± 1.0**
**33**	1711	1696 [33]	Styrallyl acetate (=1‐phenylethyl acetate)	0.30 ± 0.02	3.10 ± 0.10	2.60 ± 0.20
**34**	1722	1685–1732 [30]	Dodecanal	0.80 ± 0.02	—	—
**35**	1792	1730 [40]	4‐*tert*‐Butylcyclohexanol^d^	3.00 ± 0.10	—	—
**36**	1838	1789–1842 [30]	(*E*)‐*β*‐Damascenone	0.40 ± 0.00	—	—
**37**	1845	1802–1846 [30]	(*E*)‐Anethole	—	0.40 ± 0.02	—
**38**	1868	1820–1843 [30]	(*E*)‐Geranyl acetone	1.80 ± 0.01	—	—
**39**	1958	1892–1958 [30]	(E)‐*β*‐Ionone	2.10 ± 0.18	—	—
**40**	1999	1989 [41]	*trans*‐*β*‐Ionone‐5,6‐epoxide	0.20 ± 0.00	—	—
**41**	2021	2026 [42]	Diphenyl oxide (=diphenyl ether)	**5.20 ± 0.40**	**9.70 ± 0.70**	**9.00 ± 0.20**
**42**	2047	2037 [33]	Isoamyl salicylate	1.20 ± 0.08	0.70 ± 0.02	—
**43**	2112	2097 [43]	Amyl salicylate (=Benzoic acid, 2‐hydroxy‐, pentyl ester)	1.20 ± 0.00	1.10 ± 0.08	—
**44**	2133	2106 [44]	Phenoxy ethyl isobutyrate^d^	0.60 ± 0.02	—	—
**45**	2179	2179 [45]	3,4‐Dimethyl‐5‐pentylidene‐2(5H)‐furanone	0.50 ± 0.08	—	—
**46**	2193	2135 [46]	Methoxynaphthalene	0.80 ± 0.08	1.60 ± 0.03	—
			**Total**	**93.20**	**91.20**	**80.80**

t, trace (<0.1%).

^a^ RRI, relative retention indices calculated against *n*‐alkanes.

^b^ RRI, relative retention indices reported in literature.

^c^ Relative peak area percentages of each compound after SPME extraction.

^d^ Tentatively identified.

The most complex leaf profile was characterized by aldehydes, including several lipid‐derived aldehydes, accounting for 47.8% of the total relative peak area, with phenylacetaldehyde (13.3%), (E,Z)‐2,4‐heptadienal (10.6%), (E,E)‐2,4‐heptadienal (7.6%), diphenyl ether (5.2%), and nonanal (4.1%) as the main constituents. The aldehyde‐rich composition is consistent with previous investigations on *Lactuca* species, particularly *L. serriola* L. and *L. sativa* L., where these compounds represent a prominent fraction of the leaf volatilome [15, 16, 17]. It is also associated with the activity of the lipoxygenase pathway, which produces green leaf volatiles involved in defence, wound signalling, responses to abiotic stress, and plant–plant communication [47, 48, 49]. The observed profile reflects the active physiological role of leaves as the primary interface between the plant and its surrounding environment [47, 48, 49].

The root profile, displaying a distinct chemical pattern, was marked by a relatively higher contribution of oxygenated monoterpenes and monoterpene hydrocarbons, including *trans*‐chrysanthenyl acetate (8.1%), linalool (5.1%), *p*‐cymene (4.9%), and 1,8‐cineole (3.5%), together with hexanal (18.0%), acetophenone (13.4%), and diphenyl ether (9.7%) as the main compounds. These VOCs have frequently been implicated in belowground ecological interactions, such as communication with soil microorganisms, defence against soil‐borne pathogens and herbivores, and modulation of rhizosphere communities [50, 51].

The seed profile was the simplest, with the identified compounds accounting for 80.8% of the total relative peak area. Its composition was dominated by aldehydes and ketones, particularly nonanal (16.3%), acetophenone (15.1%), diphenyl ether (9.0%), and linalool (8.9%). Compared with vegetative organs, seeds nevertheless showed a relatively high abundance of lipid‐derived molecules. Similar patterns have been described in *L. sativa* and other taxa, where aldehydes generated through fatty acid oxidation contribute substantially to seed volatile composition [16, 52]. The presence of aromatic compounds such as acetophenone and diphenyl ether further distinguishes the chemical profile of reproductive tissues, reflecting the specialized metabolism associated with their development and physiological function [52].

Previously reported volatile profiles of different organs of *L. serriola*, obtained using the same analytical approach [15], revealed both similarities and differences with those observed in *L. tatarica*. Phenylacetaldehyde, nonanal, limonene, *p*‐cymene, linalool, 2,4‐heptadienals, *β*‐damascenone, and geranyl acetone have also been reported in other *Lactuca* species [15, 16, 17], suggesting the occurrence of a common volatile repertoire within the genus. However, their relative abundances varied substantially among the investigated organs. Phenylacetaldehyde was the predominant constituent only in leaves, whereas nonanal reached its highest abundance in seeds and linalool was comparatively enriched in roots and seeds. Moreover, *trans*‐chrysanthenyl acetate was detected exclusively in the roots, further emphasizing the organ‐specific distribution of VOCs in *L. tatarica*. These findings, obtained from air‐dried plant material, indicate that, although several volatile compounds are shared within the genus, their distribution is influenced by both species‐ and organ‐specific factors, emphasizing the importance of organ‐resolved analyses for a more comprehensive understanding of the *Lactuca* volatilome [53], while recognizing that the profiles may differ from those of fresh plant material.

## 3 Conclusions

The present study demonstrates clear metabolic differentiation in the volatile composition of *L. tatarica* organs. The occurrence of both shared and organ‐specific compounds, together with differences in their relative abundance, provides further insight into the chemical characteristics of this species. Comparison with previously reported *Lactuca* profiles indicates the presence of a common volatile repertoire within the genus, while also highlighting the variability of its composition. Overall, these findings expand the phytochemical knowledge of *L. tatarica* and support the value of organ‐resolved approaches for comparative investigations within the genus.

## 4 Experimental Section

### 4.1 Plant Material

The investigated parts of *L. tatarica* (roots, leaves, and seeds) were collected during summer 2022 from Novkhany village, Absheron district, Azerbaijan (40°34′31.27″ N, 49°46′17.91″ E; 27 m below sea level). The plant material was identified by E.N. Shukurlu according to the *Flora of Azerbaijan* [2], and voucher specimens (BAK19034 and BAK19035) were deposited at the Institute of Botany, Baku, Azerbaijan. Prior to analysis, all samples were air‐dried at room temperature away from direct sunlight.

### 4.2 Chemicals

*n‐*Hexane was purchased from Sigma‐Aldrich (St. Louis, MO, USA). A C_8_–C_40_ *n*‐alkane standard solution was obtained from Fluka (Buchs, Switzerland). A manual SPME holder (57330‐U, Supelco, Bellefonte, PA, USA) equipped with a PDMS–DVB fiber (65 µm, blue type) was used for volatile extraction.

### 4.3 MSD–SPME Extraction of Volatile Compounds

Volatile compounds were extracted using MSD–SPME as previously described [27, 28, 29]. Briefly, 0.5 g of ground plant material was placed in a 25 mL round‐bottom flask with 3 mL of distilled water. The system was equipped with a Claisen distillation head fitted with a condenser for reflux and a threaded plug accommodating the SPME fiber.

Prior to extraction, the PDMS–DVB fiber was conditioned at 250°C for 10 min. The fiber was exposed to the headspace once boiling commenced, and extraction was carried out for 2 min. After sampling, the fiber was retracted and immediately transferred to the GC injector, where thermal desorption was performed at 250°C for 5 min. Three independent extractions were performed for each sample, and results are expressed as mean values.

### 4.4 GC–MS Analysis

GC–MS analysis was carried out using an Agilent 5975 GC–MSD system (Agilent Technologies, Santa Clara, CA, USA) as previously reported [29]. Separation was achieved on an Innowax FSC column (60 m × 0.25 mm, 0.25 µm film thickness) using helium as carrier gas (0.8 mL/min).

The oven temperature was programmed from 60°C (10 min) to 220°C at 4°C/min (held for 10 min), then increased to 240°C at 1°C/min. The injector temperature was set at 250°C with a split ratio of 40:1. Mass spectra were recorded at 70 eV over the *m/z* range 35–450.

### 4.5 GC–FID Analysis

GC–FID analysis was carried out on an Agilent 6890N GC system under the same chromatographic conditions as GC–MS. The effluent was split between MS and FID detectors. The FID temperature was set at 300°C.

### 4.6 Identification of Volatile Components

Compounds were identified by comparison of their mass spectra with those from the Wiley–NIST GC/MS Library (Wiley, NY, USA) [26], MassFinder 4.0 (Dr. Hochmuth Scientific Consulting, Hamburg, Germany) [27], and the Adams Library [28], and by comparison with literature data. Whenever possible, identifications were confirmed by co‐injection with authentic standards. Additional confirmation was obtained using the in‐house “Başer Library of Essential Oil Constituents.”

Relative retention indices (RRI) were calculated using a C_8_–C_40_ *n*‐alkane standard mixture under the same chromatographic conditions. Relative peak area percentages were calculated from GC–FID peak areas after SPME extraction.

## Author Contributions

All authors have accepted responsibility for the entire content of this submitted manuscript and approved its submission.

## Conflicts of Interest

The authors declare no conflicts of interest.

## Data Availability

The data that support the findings of this study are available from the corresponding author upon reasonable request.
